# Transition clinics in pediatric rheumatology in Colombia: reflection on a necessary shortcomings

**DOI:** 10.1186/s42358-024-00419-2

**Published:** 2025-01-02

**Authors:** Lauren Natalia Ramirez, María Elisa Hoyos, Angela Catalina Mosquera-Pongutá, Gerardo Quintana-López

**Affiliations:** 1 Grupo de Investigación Reumavance, Facultad de Medicina, Universidad Nacional de Colombia, Bogotá, Colombia; 2 Grupo de Investigación Reumavance, Facultad de Medicina, Universidad de los Andes, Bogotá, Colombia; 3 Grupo de Investigación Reumavance, Especialista en Reumatología Pediátrica, Hospital Universitario Santa Fe de Bogotá, Clínica Infantil Colsubsidio y Clínica Santa María del Lago, Bogotá, Colombia; 4 Grupo de Investigación Reumavance. Sección de Reumatología, Departamento de Medicina Interna, Hospital Universitario Santa Fe de Bogotá, Bogotá, Colombia; 5 Rheumatology Section, Department of Internal Medicine, Fundación Santa Fe de Bogotá University Hospital, Carrera 7 No. 117-15, Bogota, Colombia

**Keywords:** Transition clinics, Rheumatology, Pediatrics

## Abstract

**Introduction:**

Transition clinics are conceived as programs dedicated to the active, multidimensional development of a process that addresses the medical, psychosocial, educational, and vocational needs of pediatric patients suffering from a chronic disease that will persist into adulthood. Their understanding is justified in physiological, psychological, and sociocultural terms on the basis of the differential morbidity and mortality associated with a chronic disease that begins in childhood and prevails into adulthood.

**Materials and methods:**

Here, we reflect on the history, structure, and impact of transition clinics in pediatrics, with an emphasis on pediatric rheumatologic diseases. Additionally, we propose comprehensive reflection as an alternative for the patient, their family, and the medical team, outlining guidelines for development, implementation, and evaluation.

**Results:**

The transition of care should commence in early adolescence, considering each patient’s cognitive ability as a condition for the initiation of an educational process involving introspection into the disease. Interdisciplinarity is defined as a team that addresses the clinical, physical, emotional, and social dimensions of each patient and their interaction with the environment within the framework of individualized care and family support. Despite this, the lack of evidence supporting standardized guidelines for the implementation and overall effectiveness evaluation of these interventions was highlighted.

**Conclusions:**

The transition process is considered successful when the patient is adherent and has a positive and informed perception of their health‒disease journey. We urge the generation of evidence documenting the comprehensiveness of processes inherent to transition clinics as the foundation of necessity.

## Introduction

**Fig. 1:**
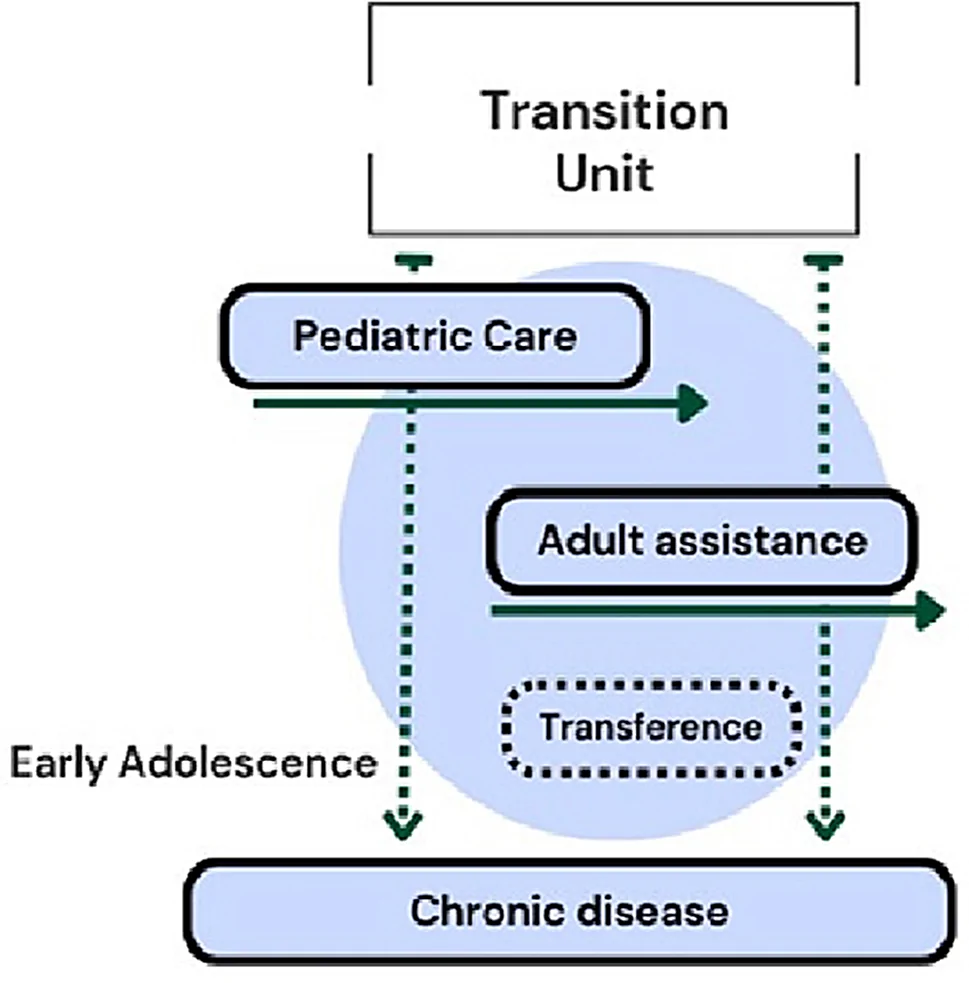
Scheme of the relationship between transition clinics and transfer to adult care

The Society for Adolescent Medicine defines transition clinics as programs or institutions dedicated to the intentional and planned transition of pediatric patients with chronic diseases to a healthcare system oriented toward adults [1]. Transition is an active, multidimensional process that addresses the medical, psychosocial, educational, and vocational needs of pediatric patients with chronic diseases that persist into adulthood [2]. In contrast, transfer is an administrative event in which the patient moves from a pediatric specialist to adult management [3]. The benefits resulting from the implementation of these programs are supported by evidence in terms of reduced morbidity and mortality [4, 5], as well as increased adherence to care and fewer associated adverse effects [5, 6] (Fig. 1).

Pediatric rheumatologic diseases (PRD) are defined as a group of autoimmune-origin, chronic, multisystem inflammatory conditions in children or adolescents [7]. PRD can become highly incapacitating, having a deleterious effect not only on patients’ quality of life by limiting their participation and psychosocial development but also on their families or caregivers, imposing emotional and economic burdens that surpass those of an adult patient [8–10], as highlighted in a published narrative review by Hersh et al., who reported that up to 20% of adults experiencing active disease since childhood are unable to work or engage in routine physical activities [9].

**Fig. 2:**
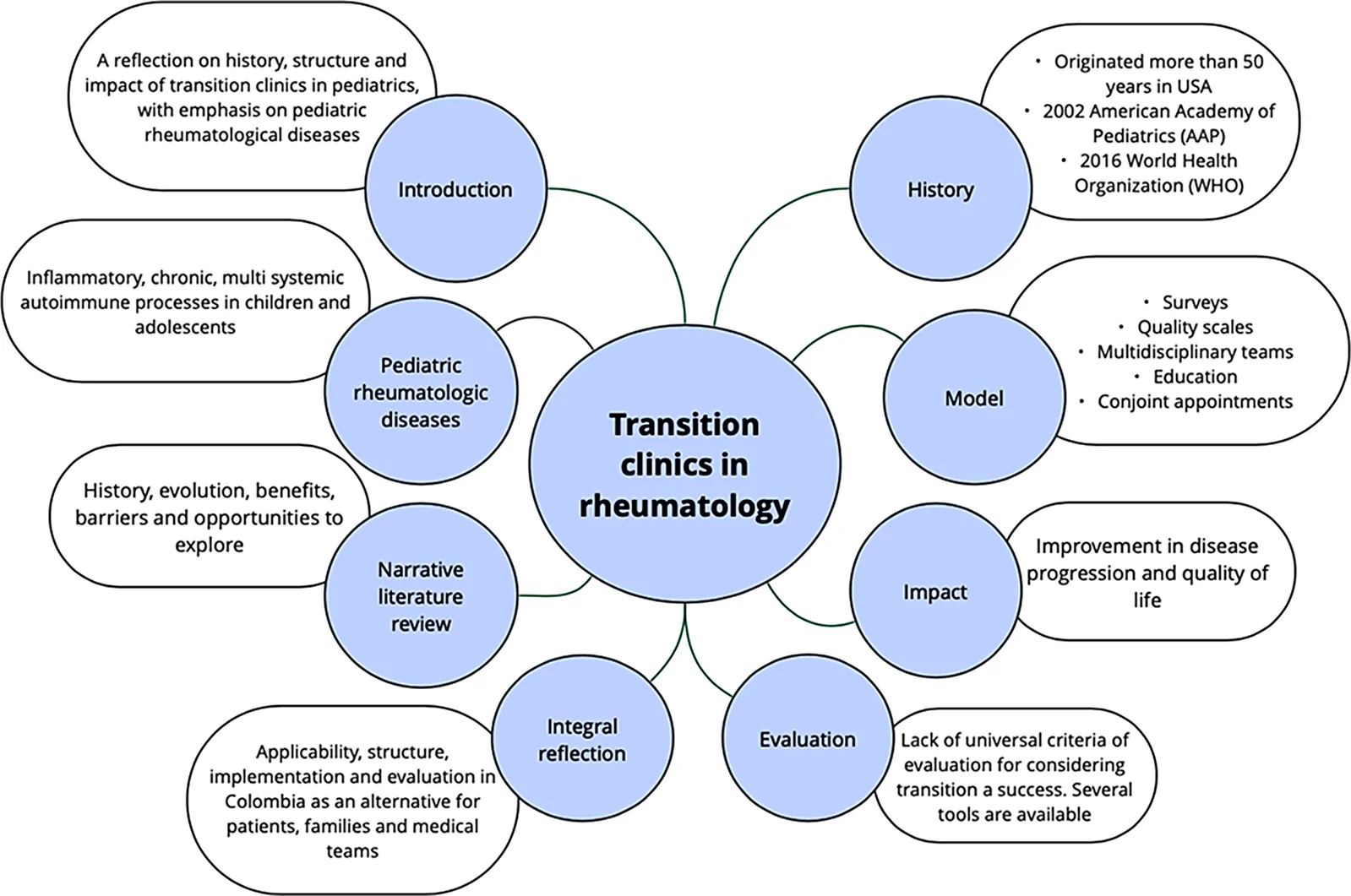
Transition clinics in pediatric rheumatology in Colombia. Content summary

Here, we explore, from a comprehensive perspective, the topic of transition clinics, the different proposed models, their benefits, barriers, and unexplored opportunities. The aim is to reflect on the possibility of structuring a comprehensive, implementable, and reproducible proposal in our local clinical context (Colombia), with an emphasis on pediatric rheumatology (Fig. 2).

## Methodology

This is a narrative literature review, we systematically searched databases such as Uptodate, PubMed, ScienceDirect, and the Virtual Health Library LILACS. We searched these bibliographic databases between January 1, 1990, and February 22, 2024. We included articles containing the terms “transition” or “transfer” to the adult healthcare system, focusing on patients aged 11–21 years. The types of reviewed texts included meta-analyses, randomized trials, and systematic reviews published in recent years. We described different transition clinical models, with a particular emphasis on pediatric rheumatologic diseases, covering their history, evolution, benefits, barriers, and unexplored opportunities. Finally, we propose a comprehensive reflection, understanding it as an alternative for the patient, their family, and the medical team, including guidelines for its structuring, development, implementation, and evaluation.

## Results

### Pediatric rheumatologic diseases (PRD)

Globally, it is estimated that up to 6.5% of the pediatric population is suspected or diagnosed with some chronic rheumatologic disease [11, 12]. In 2011, Pineles et al., in another systematic review, reported a prevalence of juvenile systemic lupus erythematosus (JSLE) ranging from 1.89 to 25.7 per 100,000 inhabitants, with a lack of information for Africa, Australia, and South America [13]. More recently, Dave et al., in a systematic review, reported a prevalence for 2017 of juvenile idiopathic arthritis (JIA) of approximately 2,069,246 and for JSLE of approximately 206,931 individuals under 16 years old (per 2,069,000,000 individuals under 16 years old), with a greater differential in Asia (South Asia), followed by Africa, America, Europe, and Oceania [14]. Reports on the PRD in Colombia are scarce and face reporting barriers in both urban and rural areas [15], highlighting an opportunity for standardized research, reporting, and monitoring.

As mentioned by Hersh et al., diseases such as pediatric or JSLE have a much more aggressive course in children. The convergence of the same pathophysiology with dynamic growth processes results in early-onset chronic inflammation leading to complications such as premature atherosclerosis, osteoporosis, and fertility problems. On the other hand, deleterious effects related to the drug toxicity of most immunomodulators when used from an early age are also common [9].

### Transition clinics

The Society of Adolescent Medicine defines transition clinics as “programs or institutions dedicated to the intentional and planned transition of pediatric patients with chronic diseases to an adult-oriented healthcare system” [3]. The first programs that conceived the need for a guided and multidisciplinary transition process in pediatrics took place over 50 years ago in the United States for patients with complex congenital anomalies (anorectal malformations, heart conditions) and postneonatal surgical conditions [16, 17].

In the early 1990s, the Canadian Institute of Child Health began describing and proposing transition programs that respond to the need for independence generated by patients with chronic diseases and how this changes during their growth process; for example, in patients with epilepsy, in addition to medical care, social support aimed at seeking greater autonomy is needed [18].

In 2002, the American Academy of Pediatrics (AAP) emphasized the need for “a planned transition of healthcare to maximize functioning and lifelong well-being for all youth“ [19]. Similarly, in the 2005 Bangkok conference, the World Health Organization (WHO) defined transition programs or transitional care as part of strategies linked to primary health care, directing their applicability through intersectoral management [20].

In the last two decades, transition programs have been the subject of study, with significant improvement and development. In 2011, the consensus on transition proposed two pillars for a successful transition: first, considering the home and the age range in which the patient will be prepared, and second, assessing individual needs and patient introspection through periodic follow-ups identifying behavior within their environment [21]. On the other hand, in 2016, the WHO issued guidelines on this matter, highlighting it as a necessary measure for comprehensive care for each patient [22].

### Structure

Although there is no standardized age for initiating the transition process, most studied programs recommend starting the process between 11 and 18 years of age; this age varies according to the study and the disease [23]. This limitation in the definition may be due to the age of disease onset or the process of establishing therapy and a physician in charge of chronic patients. However, on average, the age of the programs below started as early as 13 years and as late as 17 years.

There are many transition programs, mostly specific to each clinical condition they are applied to, with distinct characteristics, as presented in Table 1, “Examples of Transition Programs,” which can be used as a baseline model if one wishes to implement this initiative (Table 1).

**Table 1:** Examples of transition clinics in practice for various chronic pathologies

Program name	Disease	Place of execution	Model components
DON’T RETARD Belgium [24] (Hilderson et al., 2013)	Juvenile idiopathic arthritis.	Department of Pediatric Rheumatology, University Hospitals, Leuven, Belgium	Multidisciplinary team. Patient and family educators.
Ready, Steady, Go [25] England (Nagra et al., 2015)	This program receives children with any chronic disease (kidney, respiratory, rheumatological) from the age of 11.	National Health Service teaching hospital, Southampton Children’s Hospital, Southampton, UK	A series of questionnaires, advice and relationships with pediatric and adult management teams.
IBD Transition Clinic [26] EEUU (Williams et al., 2017)	Crohn’s Disease, Ulcerative Colitis and Indeterminate Colitis	Penn State University Milton S. Hershey Medical Center, Hershey, PA	Questionnaire and education process for management of chronic pathology
Are we there yet? [27] England	Early onset rheumatologic diseases	cohort studies and registries. Not a single place	Questionnaires and educate patients and their family and identify how ready they are
Transition Readiness in Adolescents With Juvenile Idiopathic Arthritis and Childhood-Onset Systemic Lupus Erythematosus [28] Canada	Juvenile Rheumatoid Arthritis, Sistemic Lupus Erythematosus.	Hamilton Health Sciences, CanChild Center for Childhood Disability Research at McMaster University	Academic and preparation process by a multidisciplinary team that takes into consideration individual needs.
Transition in a pediatric rheumatology unit Portugal [29]	Various Rheumatological diseases.	Santa Maria Hospital. Rheumatology and bone diseases service	Questionnaires, educational processes and appointments with both services.
Development of a clinical transition pathway for adolescents in the Netherlands [30]	Various rheumatological diseases	Tertiary care children’s hospital in the Netherlands.	Discussions, management of self-care skills and early presentation to adult service
There are two studies of: 1. Case report: Importance of pediatric rheumatologists and transitional care for juvenile idiopathic arthritis-associated uveitis: a retrospective series of 9 cases [31] 2. Cohort Study: Disease activity and transition outcomes in a childhood-onset systemic lupus erythematosus cohort [32]	1. Juvenile rheumatoid arthritis associated with Uveitis 2. Lupus erythematosus	1.Department of Pediatric Rheumatology and Department of Ophthalmology at the Tokyo Medical 2. Childhood Arthritis and Rheumatology Research Alliance (CARRA)	1. Carried out a retrospective report in which they found that of 9 patients with arthritis linked to uveitis, two developed blindness due to late start of therapy due to failure in the transition process. 2. Reported that patients with a longer time to obtain a treatment service would have an SDAI with greater disease activity and saw an association between anxiety and depression in the patients.
DON’T RETARD Belgium [33]	Juvenile idiopathic arthritis.	Department of Pediatric Rheumatology, University Hospitals, Leuven, Belgium	Multidisciplinary team. Patient and family educators.
Ready, Steady, Go England [34]	Children with any chronic disease (kidney, respiratory, rheumatological) from the age of 11.	National Health Service Teaching Hospital, Southampton Children’s Hospital, Southampton, UK	A series of questionnaires, advice, and relationships with pediatric and adult management teams.
IBD Transition Clinic EEUU [35, 36]	Crohn’s Disease, Ulcerative Colitis and Indeterminate Colitis	Penn State University Milton S. Hershey Medical Center, Hershey, PA	Questionnaire and education process for management of chronic pathology
Are we there yet? England [37]	Early onset rheumatologic diseases	Cohort studies and registries.	Questionnaires and educate patients and their families and identify how ready they are
Transition Readiness in Adolescents With Juvenile Idiopathic Arthritis and Childhood-Onset Systemic Lupus Erythematosus. Canada [38]	Juvenile Rheumatoid Arthritis, Systemic Lupus Erythematosus.	Hamilton Health Sciences, CanChild Center for Childhood Disability Research at McMaster University	Academic and preparation process by a multidisciplinary team that takes into consideration individual needs.
Transition in a pediatric rheumatology unit Portugal [39]	Various rheumatological diseases.	Santa Maria Hospital. Rheumatology and bone diseases service	Questionnaires, educational processes, and appointments with both services.
Development of a clinical transition pathway for adolescents in the Netherlands [40]	Various rheumatological diseases	tertiary care children’s hospital in the Netherlands.	Discussions, management of self-care skills, and early presentation to adult service
There are two studies: 3. Case report: Importance of pediatric rheumatologists and transitional care for juvenile idiopathic arthritis-associated uveitis: a retrospective series of 9 cases [24] 4. Cohort Study: Disease activity and transition outcomes in a childhood-onset systemic lupus erythematosus cohort [25]	3. Juvenile rheumatoid arthritis associated with Uveitis 4. lupus erythematosus	1. Department of Pediatric Rheumatology and Department of Ophthalmology at the Tokyo Medical 2. Childhood Arthritis and Rheumatology Research Alliance (CARRA)	1. They carried out a retrospective report in which they found that of 9 patients with arthritis linked to uveitis, two developed blindness due to the late start of therapy due to failure in the transition process. 2. They report that patients with a longer time to obtain a treatment service would have an SDAI with greater disease activity and saw an association between anxiety and depression in the patients.

According to the transition clinic for tuberous sclerosis by Peron, A., there is a focus on the 4 Ws (the four Ws):

Who: Various literature reviews, cohort studies, and case reports exist on this topic. Healthcare professionals (specialized in public health, pediatrics, pediatric endocrinology, pulmonology, child neuropsychiatry, pediatric surgery, internal medicine, endocrinology, diabetes specialists, and neurology), pediatric and adult nursing, psychology, and social work should initiate discussions about the transition process with patients [41].

When: The effective transfer from the pediatric to the adult environment is planned at 18 years of age or as soon as the patient demonstrates sufficient autonomy in managing the disease.

What: This multidisciplinary group, which acts as a Delphi panel within the healthcare system, will hold approximately 20 sessions to define the transition clinic model to be implemented in the healthcare institution.

Where: A concise explanatory document about the applicable transition model for the chronic disease under study should be developed and published on the hospital’s website for patients and their families. It will be determined whether the healthcare institution will transform into a joint clinic [42, 43].

Second, Bert, F. et al. conducted a study in Turin proposing an organizational model for transition, differentiating steps on the basis of patient complexity. The patient clearly requires a multidisciplinary transition team consisting of or including medical professionals (public health experts, pediatricians, pediatric endocrinologists, diabetes specialists, and pediatric neurologists), adult and pediatric nursing staff, psychologists and social workers. In this way, by acting as a Delphi group, they discussed the most appropriate transition model adopted by the hospital. The working group defined a standardized transition scheme, explained through shared transition programs with all involved clinics. Subsequently, they created an explanatory document for patients and families published on the hospital’s official website. They established two pathways on the basis of clinical and social complexity.

### Impact

In 2016, a compilation of Cochrane information was conducted by Campbell et al., encompassing randomized clinical trials, controlled before-and-after studies (CBAs), and interrupted time series studies. The evaluation focused on the effectiveness of interventions aimed at improving the transition of care from pediatric to adult services for adolescents with ongoing clinical care needs. The conditions examined included diabetes mellitus, cystic fibrosis, muscular dystrophy, juvenile idiopathic arthritis (JIA), congenital heart disease, cerebral palsy, autism, solid organ transplantation, and epilepsy. These studies involved patients completing their care in pediatric services without age restrictions. Interventions aimed at enhancing transition, dedicated adolescent units, joint clinics, and the use of transition-specialized personnel were employed. These interventions included clinimetrics, guided educational processes, and individual and group therapy for the involved patients [1].

The study delved into the appropriate age for initiating the transition process, the inherent factors related to the shift in the healthcare system, and the lack of knowledge among patients and their families regarding the disease and the new healthcare regimen. It was concluded that a preferable scenario involves the patient being part of a single institution or joint clinic where the transition can be systematically planned. Furthermore, the importance of healthcare personnel education has been highlighted.

The impact on clinical, psychological, and therapeutic outcomes in pediatric rheumatologic transition clinics has been extensively discussed in multiple studies [44]. One example is the study titled “Disease Activity and Transition Outcomes in a Childhood-onset Systemic Lupus Erythematosus Cohort” and “Transition of Pediatric Patients with an Autoinflammatory Disease: An Alternative Version of the Daedalus and Icarus Myth.“ [45] These studies suggest that the impact should be measured on the basis of the continuity of healthcare received by the patient during the transition. The outcome was significantly better when there were no gaps in medical care or unplanned use of health services during the transition [46, 47].

In this study, sociodemographic and clinical variables were identified, and their associations with the number of missed appointments by patients were examined. An important finding is that the transition was suboptimal in patients with lower educational levels and white races, helping to identify an at-risk population requiring more support during the transition process. The time between the last pediatric visit and the first adult visit was reduced to 9 months in patients with optimized transitions. There has been long-term improvement in disease activity control. Additionally, the importance of regular check-ups is emphasized.

Finally, in “Building a transitional care checklist in rheumatology: A Delphi-like survey”, a survey was created that could be applied to any population regardless of the disease or country of origin [48].

In Walker et al.’s “Development of a Clinical Transition Pathway for Adolescents in the Netherlands”, and Bert et al.’s “Transitional Care: A New Model of Care from Young Age to adulthood”, clear objectives were established to create a transition guide applicable to any adolescent [41, 49]. Surveys and quality-of-life scales were fundamental components of the process. Additionally, simple tasks were assigned to the adolescents and their families to exercise their self-care skills. Topics such as sexuality, recreational drugs, alcohol, vocation, and hobbies were included to provide a more comprehensive approach to adolescents’ lives. On the basis of the responses, an individualized plan was developed, a coordinator was assigned, and early initiation and joint consultations were conducted. Finally, the participants were divided into two groups on the basis of case complexity, and both received multidisciplinary management from different professionals. The characteristics that differentiated the more intensive approach included socioeconomic conditions, neurodevelopmental disorders or cognitive deficits, high disease activity, and challenging pharmacological management. Both groups showed significant improvement compared with young adults who did not undergo any transition or preparation process.

Regardless of the chosen transition model, standardized regulations for each chronic disease must be in place to ensure continuity of patient care and adherence to it. The goal is to provide patients with appropriate medical care, ensuring uninterrupted care from adolescence to adulthood [42].

## Discussion and reflection

### Implementation

In the case of pediatric patients with rheumatological diseases, the concept of transition becomes relevant, considering that most patients will reach adulthood when the disease is still active. Many patients will require ongoing treatment or management of significant consequences into adulthood, and there is evidence of physical and psychosocial deterioration in those who experienced rheumatological diseases at an early age upon reaching adulthood, especially when the onset of the disease is earlier and more aggressive [50].

The prospect of a longer life expectancy poses challenges for children with chronic diseases, as they reach adolescence and transition to adult medical care. It is expected that, upon reaching this stage, they take on responsibilities related to their care and have the necessary skills to handle competencies such as self-care, introspection, and building a support network related to the disease and the healthcare system. Importantly, these responsibilities do not fall solely on the individual now considered an “adult” but also on healthcare professionals, who may not be fully aware of the complexity involved in caring for this “new adult” with a chronic disease [51, 52].

Another significant gap explained in “Mind the Gap: Improving Care in Pediatric-to-Adult Rheumatology Transition Through Education” is the evident reluctance that hinders an efficient transition process. Most pediatricians feel uncomfortable treating adolescents, and similarly, adult internists or physicians often feel that they lack the tools or patience to address patients diagnosed with diseases in childhood. Hence, the importance of educating not only patients but also professionals involved in the transition is emphasized [53].

The challenge for researchers is to establish a mechanism for evaluating transition programs that include both patient satisfaction and clinical outcomes in an easily applicable assessment for different diseases. A vocabulary describing the types of transitions should be developed, and the results should be assessed in terms of clinical interpretation, healthcare system costs, and patient satisfaction. Compared with basic transition services, it becomes easier to determine which patients would benefit most from intensive services [54]. Notably, despite the primary motivation to implement these programs to improve patients’ quality of life, evaluating disease activity seems to take a back seat.

### Transition clinics in Colombia

In Colombia, several initiatives have been undertaken and are described below:

In mid-2009, Dr. Malagón and Dr. Vargas proposed the implementation of a similar program for patients with juvenile idiopathic arthritis. The core of the proposal involves conducting four educational workshops for patients about the transition to adult care. In a preliminary phase, the expectations and concerns of both patients and their parents regarding the transition were assessed. On this basis, an instrument adapted from the Transitional Readiness Assessment Questionnaire (TRAQ) questionnaire was developed (previously identified in Table 4). This instrument allows for the evaluation of the level of readiness for transition, identification of areas where the patient and their family require support and monitoring the progress in preparation for this process [33, 34].

**Fig. 3:**
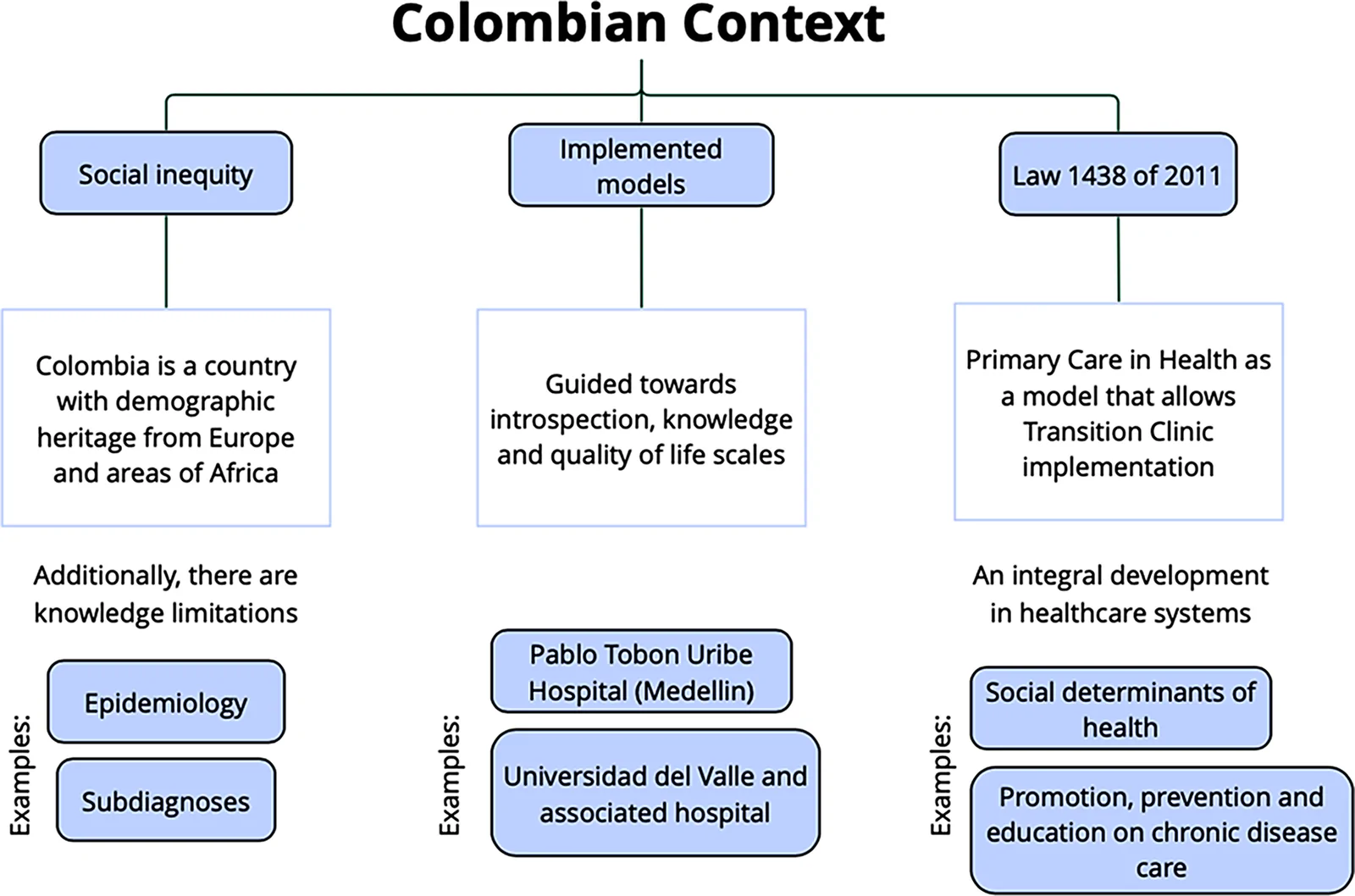
Colombian context

Similarly, at the Pablo Tobón Uribe Hospital (Medellín, Colombia) in 2018, a pilot “transition clinic” was conducted in collaboration with the University of Antioquia for patients with Lupus Erythematosus approaching adulthood. The focus was on understanding the introspection of adolescents regarding their illness as a predictor of good control. The proposed approach or intervention involves conducting surveys and counseling within the context of the perception of the disease and how to take responsibility for maintaining health [35]. A summary of the aforementioned results is provided in Fig. 3.

There is not enough national records regarding the experiences of transition clinics in Colombia or sufficient information regarding the epidemiology of rheumatological diseases [36]. Therefore, there is an opportunity for research and in-depth exploration to benefit our patients and make the approach to their pathologies more effective and beneficial [18]. Once the type of care to be provided is established, determining the vulnerable population and standardizing the model could be implemented within the framework of primary health care under Law 1438 of 2011. This could serve as a cost-effective tool for pediatric rheumatological patients, who, in the long term, will have mild disease activity, be in remission, or be better controlled, thereby avoiding hospitalizations and complications that represent an excessive cost for the healthcare system.

### Evaluation

Some alternatives reported in the literature are satisfactory references for monitoring and evaluating transition clinics. Surveys evaluating self-care tools exist. The Transitional Readiness Assessment Questionnaire (TRAQ) is a 2-question questionnaire that assesses skills and actions in two domains: self-management and self-advocacy. A higher score indicates a more positive result, with follow-ups conducted at 8 months, 6 months for self-management, and 6 months for self-advocacy. The Patient Activation Measure (PAM), a scale ranging from 0 to 100 representing an independent and functional adult with a chronic disease, is another tool. A higher score indicates a more positive result, and follow-ups are performed semiannually [1].

In studies such as Jiang et. al’s “Patient and parent perspectives on transition from pediatric to adult healthcare in rheumatic diseases: an interview”, and Kelly et.al’s “Patients’ attitudes and experiences of transition from pediatric to adult healthcare in rheumatology: a qualitative systematic review” (2020), emphasis is placed on communication failures between healthcare providers and patients, abrupt changes in management due to unfamiliarity with the patient’s medical history, the need to make patients and their families feel secure and comfortable with the new healthcare team, and reducing parental anxiety without limiting adolescent autonomy [37, 38].

In “Development and validation of the RACER (Readiness for Adult Care in Rheumatology) transition instrument in youth with juvenile idiopathic arthritis,” a validated tool was developed for patients with juvenile arthritis. It validates not only the preparedness, knowledge, and self-management of the patient and their family but also how qualified healthcare professionals are to address the intrinsic needs of the transition [39]. This study is recent (2021), and further validation in other languages and larger populations is still needed.

Despite the information presented thus far, many clinics around the world do not openly publish information, and there is no uniformity in evaluation criteria to consider the transition process successful.

A meta-analysis published in February 2022 by García et al. demonstrated that, as the transition process is sustained in clinics managing adolescent pediatric patients, it eventually reduces the need for hospitalizations, surgeries, and medication-related toxicity. Additionally, compared with the initial assessment, there was a clear improvement in the use of surveys and satisfaction questionnaires in the personal life process [40].

The results of the meta-analysis revealed a reduction in hospital admission rates (OR 0.28; 95% CI: 0.13–0.61; I 2 = 0%; *p* = 0.97) and a reduction in surgery rates in the group that received a transition program (OR 0.26; 95% CI 0.12–0.59; I 2 = 0%; *p* = 0.50). Regarding drug toxicity, no difference was observed between the groups (OR 0.61; 95% CI 0.13 to 2.83; I 2 = 29%; *p* = 0.25). Disease activity could not be analyzed because few studies reported these data [40].

## Conclusions

Transition clinics have been widely implemented worldwide; several pediatric and adult care organizations and academies have recommended their use on the basis of evidence collected in various programs demonstrating their effectiveness and considering them necessary tools in terms of primary health care. These programs have been strengthened over the years. However, there is no single approach to the transition process; below, we discuss some of these experiences.

Successful transition requires both adequate transfer to adult-centered care and the acquisition of independence and self-care of the disease by the patient. Defining a successful transition on the basis solely of clinical parameters is challenging, as there can be complex chronic diseases. Therefore, success is more focused on patient response outcomes, such as their confidence in the new system and ability to maintain their autonomy. It is necessary to rely on the assessment tools available.

### Reflection

The applicability of any alternative or technology in healthcare should be conditioned by a dynamic capacity for evaluation, development, and improvement. A satisfactory transition process involves good patient adherence to the program’s comprehensiveness and uninterrupted control, which is supervised in the form of handover with the treating team, reflecting a positive and informed perception of each patient and their family of their own health/disease process. It is also necessary to consistently evaluate disease activity as a clinical function marker, with the main goal of maintaining stability or reducing it, which is ultimately reflected in the patient’s quality of life.

Notably, each country and region has specific socioeconomic characteristics and healthcare system specificities. In Colombia, there is a lack of knowledge regarding epidemiology, subdiagnosis of diseases, and limited resources. Therefore, applying quality of life and disease activity scales requires constant and frequent evaluation of patients’ health status, which can be challenging due to their socioeconomic and geographical situation. Even in patients with continuous access to rheumatology consultations, evaluating quality of life and preparation for autonomously managing the underlying disease via routine clinical measurement is difficult owing to time constraints, and satisfaction questionnaires should be adapted and validated.

### Proposal

Given that life expectations have changed, we need to change our care model by implementing multidisciplinary strategies that adapt to the needs of patients and their caregivers. This transition improves their quality of life and facilitates their adaptation to society, which ultimately reduces costs in the provision of health services.

Therefore, we propose the following: first, clarify the epidemiological profile of rheumatic diseases in Colombia, and second, develop transition programs within the country, starting with patients who regularly attend rheumatology appointments. The validation of pediatric satisfaction questionnaires in Spanish would be necessary initially. The pilot program should include the treating pediatrician, pediatric rheumatologist, psychologist, adult rheumatologist, and a social worker or occupational therapist responsible for individualizing the processes for each patient. Depending on the disease, dynamic educational materials should be available to help patients understand their underlying disease and how their self-care processes should be. There should be open communication among all professionals responsible for the patient’s process.

We could use software or a virtual platform that allows all treating healthcare professionals to access patients’ comprehensive information (medical history, including paraclinical data, quality of life scales, and psychological profiles) and plan the transition from the pediatrician to the internist from an early age. In this way, we can prepare patients to address their needs.

Finally, we want to emphasize the importance of research opportunities in epidemiology and initiatives based on international and national experiences to improve the care received by patients with rheumatic diseases and, in the long run, reduce costs to the healthcare system, family environment, and personal aspects of adolescents.

## Data Availability

Not applicable. However, all the sources supporting our manuscript can be provided on request.
